# Influence of Different Forest System Management Practices on Leaf Litter Decomposition Rates, Nutrient Dynamics and the Activity of Ligninolytic Enzymes: A Case Study from Central European Forests

**DOI:** 10.1371/journal.pone.0093700

**Published:** 2014-04-03

**Authors:** Witoon Purahong, Danuta Kapturska, Marek J. Pecyna, Elke Schulz, Michael Schloter, François Buscot, Martin Hofrichter, Dirk Krüger

**Affiliations:** 1 UFZ-Helmholtz Centre for Environmental Research, Department of Soil Ecology, Halle (Saale), Germany; 2 Chair for Soil Science, Technical University of Munich, Oberschleissheim, Germany; 3 Department of Bio- and Environmental Sciences, International Institute Zittau, Technische Universität Dresden, Zittau, Germany; 4 Research Unit for Environmental Genomics, Helmholtz Zentrum München, Oberschleissheim, Germany; 5 German Centre for Integrative Biodiversity Research (iDiv) Halle - Jena - Leipzig, Leipzig, Germany; Wageningen University, Netherlands

## Abstract

Leaf litter decomposition is the key ecological process that determines the sustainability of managed forest ecosystems, however very few studies hitherto have investigated this process with respect to silvicultural management practices. The aims of the present study were to investigate the effects of forest management practices on leaf litter decomposition rates, nutrient dynamics (C, N, Mg, K, Ca, P) and the activity of ligninolytic enzymes. We approached these questions using a 473 day long litterbag experiment. We found that age-class beech and spruce forests (high forest management intensity) had significantly higher decomposition rates and nutrient release (most nutrients) than unmanaged deciduous forest reserves (*P*<0.05). The site with near-to-nature forest management (low forest management intensity) exhibited no significant differences in litter decomposition rate, C release, lignin decomposition, and C/N, lignin/N and ligninolytic enzyme patterns compared to the unmanaged deciduous forest reserves, but most nutrient dynamics examined in this study were significantly faster under such near-to-nature forest management practices. Analyzing the activities of ligninolytic enzymes provided evidence that different forest system management practices affect litter decomposition by changing microbial enzyme activities, at least over the investigated time frame of 473 days (laccase, *P*<0.0001; manganese peroxidase (MnP), *P* = 0.0260). Our results also indicate that lignin decomposition is the rate limiting step in leaf litter decomposition and that MnP is one of the key oxidative enzymes of litter degradation. We demonstrate here that forest system management practices can significantly affect important ecological processes and services such as decomposition and nutrient cycling.

## Introduction

Almost one-third of the world’s total forest area (ca. 1.2 billion ha) is managed primarily for wood biomass production [1]. This figure is even higher in European forests (excluding those in the Russian Federation), where 57% of the total cover is managed for wood production [1]. Forest system management practices include the forest management itself, but also conversion from one forest type to another [2], [3]. Conversion of forests includes the maintenance of non-autochthonous forests in Central Europe, for example, where European beech dominated forests were converted to age-class Norway spruce or Scots pine forests [4]. Forest management and conversion result in a decline of “naturalness” and an increase in the “artificialness” of forest ecosystems [5] e.g. from natural to semi-natural (natural regeneration) and to artificial regeneration systems (planted forest). Therefore, shifts in forest system management practices can change tree species composition and richness, stand density and age structure, either slowly over a number of decades, or perhaps suddenly because of clear-felling or burning of old growth stands [6], [7].

Although forest system management practices have the potential to affect most characteristics, compositions and structures of the entire forest ecosystem, effects on ecological processes in soils are still unclear [2], [7], [8]. For example thinning and pruning operations, which are common in intensively managed forests, have been found to affect microclimatic conditions such as light penetration, air movement and temperature [9] and therefore should also influence abiotic and biotic soil properties. Even more pronounced changes can be expected if, as a result of conversion of forest type, dominant tree species are replaced; this will influence the diversity and community structure of both above- and below-ground biota, which interact with the plants [7], [10].

Decomposition of plant litter is a complex ecological process that is regulated by three main drivers: climate, litter quality and the decomposer communities [11]–[13]. Litter quality (as determined by initial N, C/N ratio, lignin/N ratio, etc.) not only influences litter decomposition rate, but also the dynamics of nutrient mineralization and immobilization [13]. The nutrient dynamics directly affects the concentrations of nutrient elements in litter that in turn, could affect microbial decomposer and detritivore communities [14]. Microbial decomposers directly involve with litter decomposition by secreting extracellular enzymes that degrades both readily available substrates such as sugars, starch and amino acids, and larger complex substrates such as cellulose, hemi-cellulose and lignin [15]. Some studies show that lignin decomposition is the rate limiting step in litter decomposition [16], [17], thus activities of ligninolytic enzymes may greatly influence the litter decomposition rates.

In an intensively managed forest, large fractions of woody biomass are normally removed or harvested, thus leaf litter fall remains the main source of organic plant matter input into forest soil [18]–[20]. For this reason, leaf litter decomposition is the key ecological process that determines the sustainability of managed forest ecosystems. However, despite the importance of leaf litter decomposition for semi-natural and artificially regenerated forests, very few studies have investigated this process with respect to the management practices [15], [21], which is especially pronounced in Central Europe. In addition, leaf litter decomposition is strongly influenced by seasonal climatic variations, thus all short term experiments with a duration of less than 1 year are likely to suffer from biases as a result of seasonal variability and the true effects of forest system management practices on decomposition and nutrient dynamics could not be reasonably determined [22]. Nevertheless, in the South European Mediterranean climatic zone, some unique experiments investigated the decomposition rates and nutrient cycling of two different forests (natural beech vs. planted Scots pine forests) over 2 years [12], [13]. These experiments showed that different forest management practices, with different dominant tree species, could alter decomposition rates and the annual amount of N returning to the soil [12], [13].

In this study, we aimed to examine mixed deciduous leaf litter decomposition rates and the dynamics of macronutrients (N, Mg, K, Ca, P) under different forest system management practices, including natural, near-to-nature, age-class European beech dominated deciduous forests and converted forests (from European beech to monoculture Norway spruce) by means of a litterbag experiment lasting 473 days. We also investigated the activity of microbial ligninolytic enzymes and examined whether they can help to explain the leaf litter decomposition rates and nutrient dynamics under different forest system management practices. We hypothesized that the decomposition rate of deciduous litter is lower in spruce forest than in other deciduous beech dominated forests [23]. In deciduous beech dominated forests, we expected variations according to management intensity: that leaf litter decomposition rate would be highest in unmanaged natural forest, next highest in near-to-nature forest and lowest in high management intensity age-class forest [5], [15]. Intensive forest management practices were found to negatively affect lignocellulytic enzyme activities which have been reported to be a good predictor of the litter decomposition rate [15]. The results obtained from converted forests do not directly demonstrate the sustainability of such systems, but rather increase our understanding of the efficiency of the artificial system decomposer communities to break down material derived from more natural systems in the study area.

## Materials and Methods

### Ethics Statement

Field work permits were issued by the responsible environmental offices of the Free State of Thuringia (according to § 72 BbgNatSchG).

### Study Area and Treatments

The study was carried out at four forest sites across the Hainich-Dün region in Central Germany, including the Hainich National Park and its surroundings; (about 1,300 km^2^; 51°16’N 10°47’E), part of the German Biodiversity Exploratories [24]. The forests in this region grow on soils over limestone bedrock [4]. The main soil types in the study area are Luvisol and Stagnosol [24]. The soil pH is weakly acidic (5.1±1.1; mean±SD; [25]). Thin litter layers (in the range 2–5 cm) were recorded at all four forest sites. The annual mean temperature and precipitation ranged, respectively, from 6.5–8°C and 500–800 mm [4], [24]. Deciduous forests (mainly European beech, *Fagus sylvatica* dominated) are dominant in this area, covering about 83% of the total forest area. These forests can be classified, according to management types, as unmanaged forest reserves, farmers’ forests, selectively cut forests, and age-class forests [4]. The age-class management type covers the largest forest area (around 57%). The remaining forests in this region (17%) are coniferous forests dominated by Norway spruce (*Picea abies*) [4]. All information pertaining to these forests has been described in detail by [4], [24]. In this study, four forest sites (100 m × 100 m) were selected, based on their forest system management; each site represents a treatment (the main characteristics of the selected sites are presented in table 1). All sites were located less than 30 km apart, to reduce the influence of variations in geography and climate. Within each forest site, we assigned three plots (2 m × 8 m) located on flat land; these represent three replicates. The four treatments (forest management practice) in this study were: (i) Norway spruce age-class forest (SA, planted Norway spruce forest converted from European beech forest, even-age forest structure); (ii) European beech age-class forest (BA, semi-natural forest with natural regeneration, even-age forest structure); (iii) European beech selection cutting forest (BS, near-to-nature forest management with natural regeneration, uneven-age forest structure); and (iv) unmanaged deciduous forest reserves dominated by European beech (BU, uneven-age forest structure) [4], [24]. We classified the sites on the basis of a silvicultural management intensity indicator (SMI; 0–1; 0 = lowest forest management intensity, 1 = highest forest management intensity) [26]. The SMI was low in the BU (0.09) and BS (0.14) forests but high in the SA (0.40) and BA (0.46) forests [26]. SMI was calculated as the average of a risk component (SMI_r_, the risk of stand loss, function of tree species and stand age) and a density component (SMI_d_, stand density, a function of the silvicultural regime, stand age and tree species.) [26].

**Table 1:** Main characteristics of the selected forest sites in this study.

Site characteristic	Spruce age-classforest (SA)	Beech age-classforest (BA)	Beech selection cutting forest (BS)	Beech unmanaged forest (BU)
Location	Mühlhausen	Revier Sollstedt	Revier Keula	Hainich National Park
Mean annual T(°C)	6.5–8	6.5–8	6.5–8	6.5–8
Annual rainfall (mm)	500–800	500–800	500–800	500–800
Tree species richness(higher than 5 m)	5	3	2	1
Dominant tree species	*Picea abies* (>80%)	*Fagus sylvatica* (>80%)	*Fagus sylvatica* (>90%)	*Fagus sylvatica* (∼100%)
Tree species cover∼5–10%	*Fagus sylvatica Acer* sp.,*Quercus* sp.	*Acer* sp., *Fraxinus* sp.	*Acer* sp.	–
Forest system	Planted forest	Semi-natural	Semi-natural	Natural forest reserves
Forest history	Converted from beechforest	Natural regeneration	Natural regeneration	Unmanaged forest, no wood extraction >60 yrs.
Forest age structure	Even-age (∼80 yrs.)	Even-age (∼30 yrs.)	Uneven-age	Uneven-age
SMI	High (0.40)	High (0.46)	Low (0.14)	Natural, very low (0.09)
Soil type	Stagnosol (Luvisol-Stagnosol)	Luvisols (Luvisol-Stagnosol)	Luvisols (Luvisol-Stagnosol)	Luvisols (Luvisol-Stagnosol)

### Leaf Litter Collection and Litterbag Method

At each forest site, freshly fallen leaves of deciduous species were collected from the forest floor in October, 2009. Deciduous litter collected from SA was derived from deciduous trees naturally regenerated within this site. The leaves from each site were separated according to species and air dried to constant weight at room temperature. Ten grams of local mixed leaves (representative of the litter composition of the deciduous tree species at each respective site, Table 1, 2) were placed in nylon litterbags (25 cm × 25 cm, 2 mm mesh size). It has been reported that this mesh size is sufficiently small to minimize losses of leaf litter due to breakage, but to permit the activity of aerobic microbes and other decomposers such as micro-fauna [27]–[29]. A total of 216 litterbags were used for the experiment. At the end of the litter fall period (13 November 2009), 180 litterbags were placed in a horizontal position in the upper litter horizon at the study plots (45 litterbags per management type resulting in 15 bags per plot) while 36 litterbags (9 per treatment) were retained to determine the initial dry mass (oven-dried at 105°C ≥24 hr. until constant weight), nutrient element concentrations and lignin content of the litter. Litterbags were retrieved on five sampling dates: in 2010 on 10 February (89 days after incubation commenced, DAI), 12 May (180 DAI), 24 August (284 DAI), 10 November (362 DAI) and in 2011 on 1 March (473 DAI). On each sampling occasion, a random sample of nine litterbags per treatment (three litterbags per plot) were carefully removed, each put in a separate clean plastic bag to reduce the loss of small fragments and transported on ice (0°C) to the laboratory within 4 hrs. In the laboratory, litterbags were processed immediately. The extraneous organic materials adhering to the outside of the bags were removed and the leaf litter from the three litterbags retrieved from the same plot and treatment was pooled and its wet weight determined. Thus, three composite samples from each site were obtained for each treatment and DAI and each composite sample was homogenized and subsampled for determination of dry mass, nutrient concentrations, lignin content and ligninolytic enzyme activities.

**Table 2:** Information on the litter composition for individual forest sites.

Forest management practice	Litter composition (%)			
	*Fagus sylvatica*	*Acer* sp.	*Quercus* sp.	*Fraxinus* sp.
Spruce age-class forest (SA)	70	25	5	0
Beech age-class forest (BA)	85	10	0	5
Beech selection cutting forest (BS)	90	10	0	0
Beech unmanaged forest (BU)	100	0	0	0

### Physicochemical Analyses

The dry mass of leaf litter samples was determined after oven-drying at 105°C to constant weight, i.e. for ≥24 hr. Initial and subsequent leaf litter samples from different incubation periods were analyzed for C, N, and lignin. Total C and N were determined by dry combustion at 1000°C with an Elementar Vario EL III (Hanau, Germany) elemental analyzer (DIN/ISO 10694 (Aug. 1996)). Nutrient ions were determined using inductively coupled plasma (ICP) optical emission spectrometry (ICP-OES) and mass spectrometry (ICP-MS) according to manufacturers’ specifications. The ICP-OES device “Optima 3000” (PerkinElmer Inc., Waltham, MA, USA) was calibrated using “ICP multi-element standard solution IV” (Mg, K, Ca, Mn, Fe, Cu) as well as “phosphorus ICP standard” (both from Merck, Darmstadt, Germany) in 1 mg/L and 10 mg/L concentrations. The ICP-MS “Elan DRC-e” (PerkinElmer) device was calibrated using “ICP multi-element standard solution VI” (V, Mn, Fe, Co, Cu; Merck) in 2 μg/L, 15 μg/L and 50 μg/L concentrations. Samples were derived from filtered (0.7 μm Whatman GF/F filter, GE Healthcare, Buckinghamshire, UK), aqueous extracts of 10 g coarsely ground (on dry ice) litter material from one replicate. The mean values for the three biological replicates from each sampling point were calculated and presented as μg per gram of litter dry mass. Extractions and ion determinations were undertaken once for each biological replicate, dry mass determination was performed three times.

Total lignin was obtained by summing Klason lignin (acid insoluble lignin) and acid soluble lignin [30]. Klason lignin content was determined gravimetrically as the dry mass of solids after sequential hydrolysis with sulfuric acid (72% w/w); in a second step, acid soluble lignin was measured UV-photometrically in 4% H_2_SO_4_ [31], [32]. All physico-chemical analyses were conducted in triplicate on the same subsample.

### Ligninolytic Enzyme Activities

Aliquots of all samples from 89 to 473 DAI were used to determine ligninolytic enzyme activities. Three important enzymes were chosen: laccase (EC 1.10.3.2), peroxidase (EC 1.11.1.7) and manganese peroxidase (MnP; EC 1.11.1.13) [33], [34].

Laccase and peroxidase activities were spectrophotometrically determined in 1 mL cuvettes by following the oxidation of ABTS (2,2′-azino-bis(3-ethylbenzthiazoline-6-sulphonic acid) at 420 nm (ε_420_ = 36 mM^−1^cm^−1^) in a sequential assay in 50 mM sodium citrate buffer at pH 4.5 (final concentrations: 50 mM sodium citrate buffer, pH 4.5; 0,3 mM ABTS; 100 nM H_2_O_2_; 50 μL sample in 1 mL reaction). Measurement started with the addition of ABTS, the ABTS oxidation without H_2_O_2_ (laccase activity) was recorded for some time, before H_2_O_2_ was added; the peroxidase activities were corrected by those of laccase.

Manganese peroxidase activities were specifically assayed as described previously by monitoring the formation of Mn^3+^–malonate complexes at 270 nm (ε_270_ = 11,59 mM^−1^cm^−1^, [35]) (final concentrations: 50 mM Na-malonate buffer, pH 4.5; 0.5 mM MnCl_2_, 100 nM H_2_O_2_; 50 μL sample in 1 mL reaction). Measurement started when H_2_O_2_ was added.

Samples were derived from 10-fold concentrated (“Vivaspin6” centrifugal concentrator devices with 10 kDa MWCO; Sartorius Stedim Biotech GmbH, Göttingen, Germany) aqueous extracts of 10 g coarsely ground (on dry ice) litter material from one replicate. In all cases, the mean values of triplicate determinations were calculated and expressed in international units (U) defined as the amount of enzyme that forms 1 mmol product min^−1^ under the assay conditions used; enzyme activities are given per gram of dry mass of the particular litter sample. For each extract, enzyme activity and dry mass were determined three times. All chemicals used were purchased from Sigma-Aldrich (Steinheim, Germany) and Merck (Darmstadt, Germany).

### Statistical Analysis

Total C and N were expressed as a percentage of the initial value, based on ash-free dried mass. Nutrient ions (Mg, K, Ca, P) were expressed as percentage of initial values based on dry litter mass. The expression of the N, Mg, K, Ca and P contents in the leaf litter as a percentage of the initial content allowed us to determine the net N, Mg, K, Ca and P dynamics [36]. Mass loss was expressed as the percentage of remaining dried mass compared with initial dried mass. Decomposition rates (*k*) for each forest type (treatment) were determined by fitting a single negative exponential decay equation [37] to remaining leaf litter dried mass over 1.30 years (473 days) in the form:



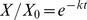



where *X* = dried mass remaining at time *t*, *X*_0_ = original mass, “*e*” = the base of natural logarithms, *k* = decomposition rate, and *t* = time (year). All data were analyzed using a one-way analysis of variance (ANOVA) incorporating the Fisher’s Least Significant Difference (LSD) implemented in SPSS software (IBM SPSS Statistics 19, New York, NY, USA) to determine the differences (*P*<0.05) between different treatments at each DAI. All datasets were tested for normality using Shapiro-Wilk and Jarque-Bera tests and the equality of group variances was examined using Levene’s test. ANOVA residuals were also plotted to determine the normal probability using Shapiro-Wilk test. In addition, ligninolytic enzyme activities were also analyzed using two-way (forest system management practice and DAI) ANOVA with *P*<0.05 considered significant, implemented in the PAST program [38].

## Results

### Effect of Forest Types on Litter Decomposition Rates

**Figure 1:**
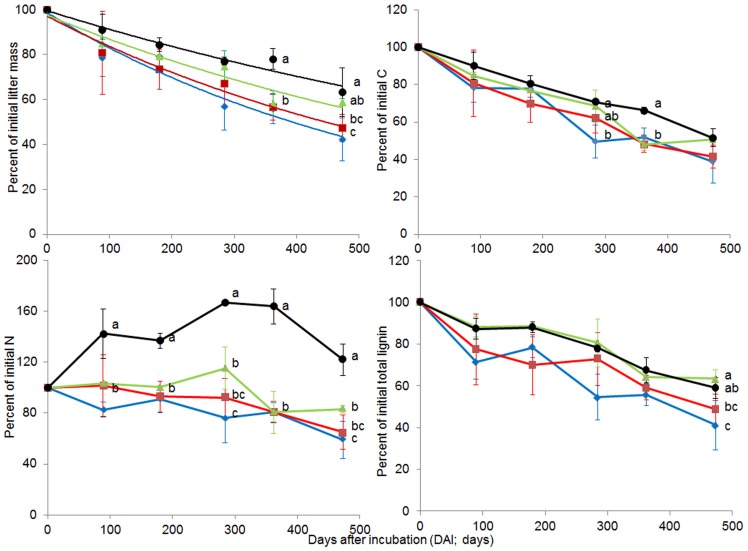
Remaining amount of leaf litter dry mass, carbon, nitrogen and total lignin during decomposition under different forest system management practices. Norway spruce age-class forest (blue, SA), European beech age-class forest (red, BA), European beech selective cut forest (green, BS) and unmanaged deciduous forest reserves dominated by European beech (black, BU) (mean ± SD, n = 3). Different letters indicate significant differences according to one-way ANOVA incorporating Fisher’s Least Significant Difference (from 89 DAI to 473 DAI).

The remaining mass of leaf litter varied significantly with forest system management practice (treatments). Surprisingly, we did not find a negative effect of high forest management intensity and/or conversion of forest types on the leaf litter decomposition rate for any of the DAI sampling times. In general, leaf litter in unmanaged beech forest (BU) decomposed relatively slower than in other forests, and the results were significant at 362 and 473 DAI (Fig. 1). One-way ANOVA demonstrated that at 362 DAI, spruce age-class (SA), beech age-class (BA) and beech selecting cutting (BS) forests had significantly lower mass remaining (higher decomposition rates) than BU (*F* = 10.37, *P* = 0.0039). At the end of the experiment, SA and BA had significantly lower mass remaining (higher decomposition rates) than BU (*F* = 4.58, *P* = 0.0378) (Fig. 1; Table 3).

**Table 3:** Mean decay rate constants (*k*), coefficients of determination (*r* ^2^) describing the fit of the decay model (*P*<0.05), and mean percentage of leaf remaining mass at the end of the study (473 days after incubation) under different forest system management practices.

Forest system management practice	*K* (year^−1^)	*r* ^2^ (*P* value)	Remaining mass (%)
Age-class spruce forest (SA)	0.64±0.12^ c^	0.96 (***P*** ** = 0.001**)	42.17±9.61^c^
Age-class beech forest (BA)	0.55±0.08^ bc^	0.98 (***P*** **<0.001**)	47.35±6.18^ bc^
Selection cutting beech forest (BS)	0.42±0.02^ ab^	0.92 (***P*** ** = 0.002**)	58.76±1.60^ ab^
Unmanaged beech forest (BU)	0.32±0.09^ a^	0.94 (***P*** ** = 0.001**)	63.29±10.89^ a^

Different lower-case letters indicate significant differences according to one-way ANOVA incorporating Fisher’s Least Significant Difference.

### Nutrient Concentrations and Effect of Forest Types on Nutrient Dynamics

Leaf litter from BU had lower amounts of N and higher C/N and total lignin/N ratios compared with other forest management practices (Table 4). Initial metal ion concentrations (V, Fe, Co, Cu) under different forest management practices were relatively similar (Table 4). All initial nutrient concentrations and other quality parameters of leaf litter under different forest management practices are listed in table 4.

**Table 4:** Initial chemical composition of dried leaf litter under different forest management practices (Mean±SD, n = 3).

Nutrient	Age-class spruceforest (SA)	Age-class beechforest (BA)	Selection cutting beechforest (BS)	Unmanaged beechforest (BU)
Total C (%)	49.05±0.19^ b^	47.61±0.17^ a^	47.34±0.16^ a^	48.81±0.22^ b^
Total N (%)	1.30±0.03^ d^	0.97±0.01^ b^	1.04±0.00^ c^	0.84±0.03^ a^
C/N	37.65±0.80^ a^	48.92±0.20^ c^	45.52±0.15^ b^	58.38±2.18^ d^
Total lignin/N	35.80±0.87^ a^	43.13±0.25^ c^	39.74±0.00^ b^	56.27±1.90^ d^
Initial Mg (μg/g dry mass)	619.33±257.02^ b^	204.00±50.27^ a^	294.33±197.28^ a^	157.93±54.19^ a^
Initial K (μg/g dry mass)	2120.00±1108.51^ a^	3076.67±545.01^ a^	4596.67±2500.11^ a^	2443.33±120.97^ a^
Initial Ca (μg/g dry mass)	1090.33±489.43^ b^	593.33±142.55^ ab^	964.33±576.11^ ab^	344.33±70.61^ a^
Initial P (μg/g dry mass)	103.87±10.74^ a^	187.67±41.50^ b^	152.67±27.43^ ab^	157.33±20.84^ a^
Initial Mn (μg/g dry mass)	69.90±33.40^ b^	32.87±9.64^ a^	26.47±11.65^ a^	20.53±5.56^ a^
Initial Fe (μg/g dry mass)	7.27±3.91^ a^	5.60±2.52^ a^	4.67±2.80^ a^	7.37±9.14^ a^
Initial Cu (μg/g dry mass)	1.27±0.22^ bc^	0.91±0.37^ b^	0.62±0.07^ ab^	0.40±0.04^ a^
Initial Co (μg/g dry mass)	0.01±0.01^ a^	0.01±0.00^ a^	0.02±0.01^ a^	0.01±0.00^ a^
Initial V (μg/g dry mass)	0.02±0.01^ a^	0.02±0.00^ a^	0.02±0.00^ a^	0.02±0.02^ a^

Different lower-case letters indicate significant differences according to one-way ANOVA incorporating Fisher’s Least Significant Difference.

**Figure 2:**
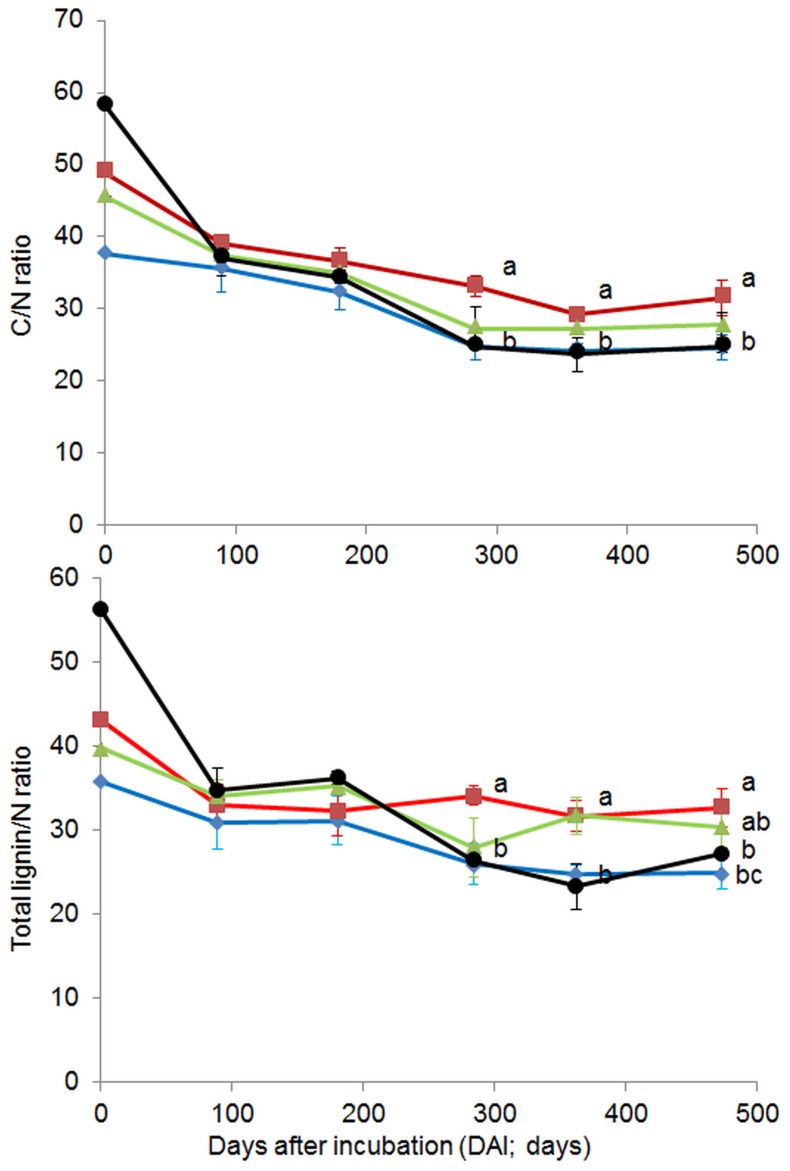
C/N and total lignin/N ratios in leaf litter during 473 days of decomposition under different forest system management practices. Norway spruce age-class forest (blue, SA), European beech age-class forest (red, BA), European beech selective cut forest (green, BS) and unmanaged deciduous forest reserves dominated by European beech (black, BU) (mean ± SD, n = 3). Different letters indicate significant differences according to one-way ANOVA incorporating Fisher’s Least Significant Difference (from 89 DAI to 473 DAI).

**Figure 3:**
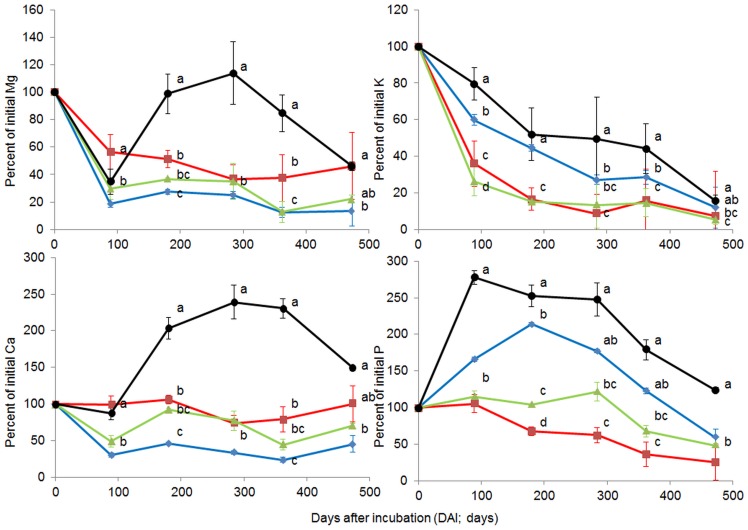
Percent of initial magnesium (Mg), potassium (K), calcium (Ca) and phosphorous (P) during decomposition under different forest system management practices. Norway spruce age-class forest (blue, SA), European beech age-class forest (red, BA), European beech selective cut forest (green, BS) and unmanaged deciduous forest reserves dominated by European beech (black, BU) (mean ± SD, n = 3). Different letters indicate significant differences according to one-way ANOVA incorporating Fisher’s Least Significant Difference (from 89 DAI to 473 DAI).

In general, the amounts of nutrients in leaf litter relative to the initial values (nutrient dynamics) were significantly influenced by forest system management practice. The changes in the amounts of different nutrients relative to their initial values, C/N and total lignin/N ratios over 473 days of incubation are shown in Figures 1, 2 and 3. For most nutrients (except C), litter from BU showed significantly slower nutrient release compared with other forest system management practices. For N and P dynamics, nutrient immobilization was clearly observed in the BU forest, where the percentage to initial values of N and P increased from the beginning of the experiment onwards, reaching peaks at 284 DAI (N = 166.49%) or 89–284 DAI (P = 277.70–247.80%) and decreasing thereafter. Similar patterns of N and P immobilizations were also observed in litter from BS, but with much lower magnitudes compared to the BU litter (N and P reached a peak at 284 DAI, with 115.27, 121.74%, respectively). Litter from SA only exhibited P immobilization and, at the first sampling date, N was released (89 DAI). In the BA forest, there were no clear patterns observed for either N or P immobilization, and N and P were released at 180 DAI. For C and K dynamics, all forests exhibited an exponential decrease from 89 DAI, however the reduced amounts of C and K varied significantly with forest system management practice (Figs. 1 and 3). Exponential nutrient release was also found for Mg and Ca under SA, BA and BS forest management practices but not under BU, where Mg and Ca had decreased at 89 DAI, increased at 180 DAI, reached a peak at 284 DAI (Mg = 113.88%, Ca = 239.21%) and decreased thereafter. The C/N ratio of leaf litter in different forests showed a similar trend: the C/N ratio decreased until 362 DAI then remained constant or slightly increased at 473 DAI (Fig. 2). At 89 DAI, the C/N ratios of all forest types were similar (*F* = 1.25, *P* = 0.3545). The BA forest had a significantly higher C/N ratio in leaf litter compared with other forests from 284 DAI until the end of the experiment (Fig. 2).

### Effect of Forest System Management Practices on Lignin Decomposition

Lignin amounts (both Klason and total lignin) in leaf litter relative to the initial values were significantly affected by forest system management practices. Patterns of lignin decomposition (both Klason and total lignin) between BU and BS (low forest management intensity) forests, and between BA and SA (high management intensity) forests were similar across the DAI sampling times (Fig. 1). Interestingly, across all DAIs, we found that the lignin decomposition rate (based on both Klason and total lignin) in the BU forest was never higher than in the BA and SA forests (Klason and total lignin were high in BU forest across DAI). The amount of lignin, relative to the initial values, between high management intensity (BA or SA) forests and low management intensity (BU and BS) forests was marginally significant at 180 DAI and 284 DAI (Klason lignin, *P* = 0.0500 and 0.0518) and at 180, 284 and 362 DAI (total lignin, *P* = 0.0558, 0.0541 and 0.0923, respectively; data not shown). Large fractions of lignin in the SA and BA forests were decomposed earlier than in the BU and BS forests; for example, at 89 and 180 DAI, SA and BA forest had lost 29–30% of Klason and total lignin while BU and BS forests reached this level around 362 DAI (Fig. 1). The Klason/N and total lignin/N ratios of leaf litter in different forests exhibited different patterns across the DAI (Fig. 2).

### Ligninolytic Enzyme Activities under Different Forest System Management Practices

**Figure 4:**
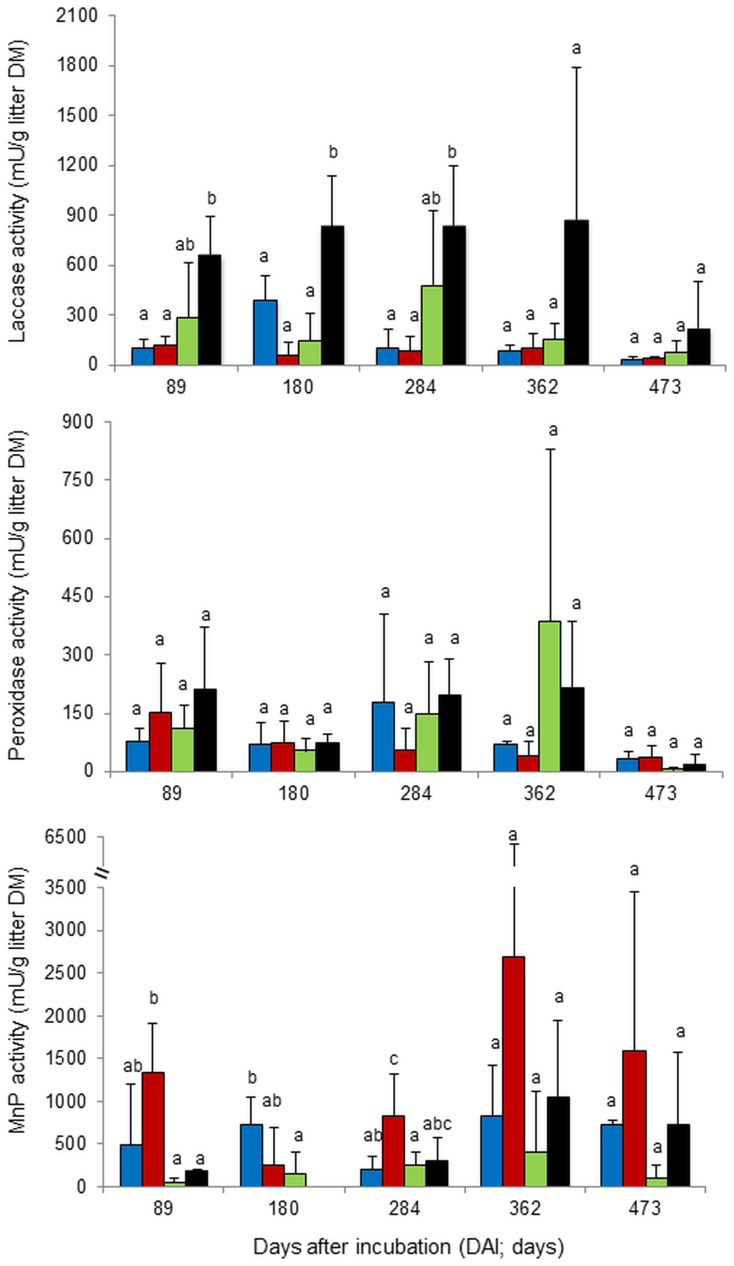
Mean ligninolytic enzyme activities in leaf litter under different forest system management practices. Norway spruce age-class forest (blue, SA), European beech age-class forest (red, BA), European beech selective cut forest (green, BS) and unmanaged deciduous forest reserves dominated by European beech (black, BU) (mean+SD, n = 3). Different letters indicate significant differences according to one-way ANOVA incorporating Fisher’s Least Significant Difference (from 89 DAI to 473 DAI). *At 180 DAI, MnP activity of unmanaged deciduous forest reserves dominated by European beech  =  0.

Different forest system management practices significantly affected laccase (*F* = 14.00; *P*<0.0001) and MnP (*F* = 3.43; *P* = 0.0260) activities, while the effects of DAI and the interaction between forest system management practice and DAI were not significant (*P*>0.05; Table 5). In contrast to these two enzymes, general peroxidase activity was significantly influenced only by time (*F* = 2.66, *P* = 0.0466), whilst the effects of forest system management practices and the interaction between forest types and time were not significant (*P*>0.05; Table 5). Forest system management practices with high and low management intensities had distinct patterns of ligninolytic enzyme activities over the incubation period (Fig. 4). SA and BA forests (high management intensity) maintained high activities of MnP at all sampling times; specifically, the MnP activities ranged from 206–830 and 251–2,694 mU/g litter dry mass in the SA and BA forests, respectively. These two forests had relatively lower laccase activities compared to the BU forest at all sampling times. On the other hand, the BU and BS forests (low management intensity) showed relatively high laccase activities but low MnP activities compared with SA and BA forests at most sampling times. Increasing MnP activity in the BU and BS forests was observed after 284 and 362 days, respectively. Considerable decreases in the remaining lignin in leaf litter of the BU and BS forests were observed after the increase in MnP activity (Figs. 1 and 4).

**Table 5:** Effects of forest system management practice, days after incubation commenced and their interactions on ligninolytic enzyme activity (P<0.05).

Factor	Laccase activity	Peroxidase activity	Manganese peroxidase activity
Forest system management practice	*F* = 14.00, ***P*** **<0.0001**	*F* = 1.13, *P* = 0.3483	*F* = 3.43, ***P*** ** = 0.0260**
Days after incubation (DAI)	*F* = 1.89, *P* = 0.1308	*F* = 2.66, ***P*** ** = 0.0466**	*F* = 1.66, *P* = 0.1780
Forest system management practice × DAI	*F* = 0.87, *P* = 0.5787	*F* = 1.09, *P* = 0.3967	*F* = 0.48, *P* = 0.9152

## Discussion

### Effect of Forest Types on Litter Decomposition Rates

The decomposition rate and the patterns of nutrient dynamics of leaf litter in the BU forest were similar to other published litterbag experiments carried out in this area [39], [40]. In our experiments we used leaf litter taken from each site individually (within each forest management practice site) to avoid deviations from the normal process of leaf litter decomposition at each particular site. It is known that senescent leaves cover microbial communities (endophytes and epiphytes) that initiate the decomposition process [41], [42]. Thus, mixing leaf litter from different sites may affect the microbial communities and their interactions; this could create results that do not represent what normally happens at each site. Therefore our study provides clear answers about how and to what extent the forest system management practices influence decomposition rates and thereby nutrient dynamics in Central European forest ecosystems. Interestingly, our results revealed that there were no significantly negative effects of intensive forest management or conversion of forest type on leaf litter decomposition and nutrient release to the forest soil. In contrast to what has been generally expected [15], [23], we found that leaf litter decomposition rates and nutrient release (most nutrients) in the converted and/or intensively managed forests (SA and BA) were significantly higher than in the unmanaged forest reserve (BU). In addition, despite having the same forest age structure (uneven-age), the decomposition rate was relatively higher and N, Ca, K and P mineralization significantly faster in BS (near-to-nature forest management) than BU forests. This may be because forest management practices alter leaf litter composition and quality. We found that leaf litter in SA, BA and BS forests was of better quality (lower C/N or lignin/N ratios) than that in BU forest (Table 4); this was due to the mix of leaf litter from other tree species (better litter quality than beech) from natural regeneration at each site (Table 1). The SA forest originated from large scale disturbance of beech dominated deciduous forest (so there has been no change in land-use, for example an agricultural period or a time as grassland) [2], thus deciduous propagules still remain in the soil and are available to regenerate naturally within the SA forest (Table 1). In beech dominated forest, the woody biomass is harvested either by whole stand harvesting (BA) or individual tree harvesting (BS) [4]. These harvests could generate the forest gaps that are important for tree regeneration and the maintenance of plant diversity [4], [43]. Such gaps tend to be nutrient rich and have relatively high light levels. In them, propagules of different plants (especially shade intolerant species) can germinate, grow quickly and maintain in the forest [43]. The unmanaged beech (BU) forest studied here is located in a National Park which, for at least 60 years, has not undergone wood harvesting or other serious disturbances that would have resulted in the loss of large numbers of trees; thus, European beech dominates the entire forest area [4]. This could explain the higher tree species richness in the BA and BS forests compared with the BU forest in our study. Due to the positive effects of tree diversity on leaf litter decomposition rate and nutrient release in managed forests, tree diversity should be maintained by allowing the natural regeneration of a range of native deciduous species, especially at the thinning stage. In this experiment, litter from the SA forest showed an interesting pattern of N and P dynamics (litter accumulates P but releases N). This may be again related to the initial leaf litter stoichiometry: initial C/N and C/P ratios. Manzoni et al. [44] reported that stoichiometry regulates carbon, nitrogen, and phosphorus dynamics in decomposing litter. In SA forest, the initial C/N ratio was low and possibly below critical ratio for N mineralization in this ecosystem while the initial C/P ratio was considerably high and does not reach the critical ratio for P mineralization [44].

Nevertheless, changes in leaf litter stoichiometry due to the higher tree richness in BA and BS forests do not explain all the effects of forest management practices on litter decomposition rates. It is unlikely that small fractions (5–10%) of leaf litter from an additional one or two tree species in BA and BS forest compared with BU forest could dramatically change the decomposition rate (31–72%). There is some evidence that forest management practices not only change tree species richness but also the microclimate and the microbial decomposer community [9], [45].

### The Role of Microbes

The higher mass loss and net nutrient release (for most of nutrients) in the BA forest as compared with the BU forest may be due to changes in the soil microbial community structure and activity induced by different forest system management practices, as reported in a recent soil fungal diversity study carried out at the same study sites [45]. Although these changes were not statistically significant for fungal communities, there was a substantial shift of 23% (121–123 fungal OTUs were unique to each forest type) when compared to fungal communities in BU and BA forests. It is reasonable to conclude that particular taxa may have substantial impact on litter degradation dynamics so that these community changes may not be so subtle in their effect.

When comparing SA and beech dominated forests, we found no significant home-field advantage. This is because the SA forest in this experiment is accustomed to periodic deciduous litter deposits. In our study, we were able to sample deciduous litter on this site simply because some deciduous trees (∼20%) have established here as a result of natural regeneration. We also postulate that because decomposer communities in SA forest can cope with more recalcitrant substances, such as resins, polyphenols and guaiacyl lignin found in spruce needles, they may also have the capacity to decompose the main deciduous leaf litter (European beech), which is also considered to be a low quality litter, quite rapidly [20].

### Lignin Degradation and Carbon Dynamic

In this study, we demonstrated that the decomposition of complex substances such as lignin was not negatively affected by forest system management practices and/or the naturalness of the forest ecosystems. We found that semi-natural and artificial systems (BA and SA forests) are more efficient at decomposing lignin than near natural (BS forest) and natural systems (BU forest). In addition, the percentage of remaining C in the leaf litter in BU forests was high (indicating slow decomposition) compared with other forest system management practices at all sampling times, and at the end of the experiment there was no significant difference in remaining C in different forest management types. Since no significantly negative effects of forest system management practices were found for either lignin or C decomposition, we can conclude that C cycling was not negatively influenced by intensive forest management, although we could not quantify the exact amounts of leaf litter C release to the forest soil, since litter C can be released both to the soil and to the atmosphere [11]. Some recent studies carried out at the same study sites have also found that forest management had no detectable effect on turnover of mineral associated soil organic matter [46] or on current soil organic carbon (SOC) stocks [47].

The ligninolytic enzyme activities recorded indicate that forest system management practices control the litter decomposition rates via changes in microbial, in the first place fungal, activities. Although both laccase (high activity in BU and BS forests) and MnP (high activity in BA and SA) can catalyze the oxidation of phenolic lignin moieties, laccase has a generally lower oxidative strength and redox potential compared to peroxidases [48]–[51]. This may explain why BS and/or BU forests had lower lignin decomposition rates compared with BA and SA forests despite the high laccase activity. Interestingly, in BU and BS forests, the sharp decrease in the remaining lignin in leaf litter was only observed after the MnP activities started to increase. This strongly indicates that MnP is a key enzyme in lignin decomposition and supports the results of other studies demonstrating the ability of MnP to catalyze the disintegration and partial mineralization of lignin [52], [53]. It can be assumed that there is a shift from microbial communities producing more laccase in BU and BS forests (low forest management intensity) to more MnP producing communities (dominated by basidiomycetes) in BA and SA forests (high forest management intensity). In BA and SA forests, where the litter decomposition rates were high, high MnP activities were also observed at all sampling times. In addition, the lignin contents (as compared to the initial amounts) decreased considerably from the first sampling date on (89 DAI). This confirms that lignin decomposition is the rate limiting step in litter decomposition [16], [17] and that MnP is one of the oxidative key enzymes of litter degradation [33]. Mechanisms on how MnP is involved in the decomposition process of lignin and other recalcitrant substances have been reviewed [33]. Based on this knowledge, we propose that, also in leaf litter, MnP oxidizes Mn^2+^ (that is present in sufficient amounts in samples of all forest sites, table 4) into highly reactive Mn^3+^ that is stabilized by secreted fungal chelators such as oxalic acid. Chelated Mn^3+^ in turn performs as a small diffusible oxidant (redox mediator) attacking preferentially phenolic lignin structures which leads to the formation of instable free radicals that tend to disintegrate spontaneously [33].

### Outlook

In our experiment, we found that the differences in management may affect litter decomposition by altering the composition of the vegetation. To improve our mechanistical understanding of this point, a common garden experiment where all litters are incubated in one place may be helpful. However, litter decomposition is not only depending on the litter material used but is also strongly impacted by the microclimatic and other environmental conditions (nutrients, water availability, etc.) [11]. Thus, such a common garden experiment will only help to figure out what is the role of litter quality but not the role of the environmental conditions present at the site.

## Conclusion

We conclude that forest system management practices can significantly influence leaf litter decomposition rates and nutrient dynamics in Central European forests. The effects of forest system management practices on litter decomposition rate and nutrient dynamics in beech dominated forests (BA, BS and BU) are complex and are controlled by a range of factors, including tree diversity, microclimatic conditions and the microbial decomposer community. In managed forest ecosystems where a large fraction of woody biomass is harvested, as is the case in BA and BS forests, the system can balance the nutrient status by increasing leaf litter decomposition rates and nutrient dynamics.
